# Network-based modular latent structure analysis

**DOI:** 10.1186/1471-2105-15-S13-S6

**Published:** 2014-11-13

**Authors:** Tianwei Yu, Yun Bai

**Affiliations:** 1 Department of Biostatistics and Bioinformatics, Rollins School of Public Health, Emory University, Atlanta, GA, USA; 2 Department of Pharmaceutical Sciences, School of Pharmacy, Philadelphia College of Osteopathic Medicine, Suwanee, GA, USA

**Keywords:** matrix decomposition, modularity, latent factors, network, community detection

## Abstract

**Background:**

High-throughput expression data, such as gene expression and metabolomics data, exhibit modular structures. Groups of features in each module follow a latent factor model, while between modules, the latent factors are quasi-independent. Recovering the latent factors can shed light on the hidden regulation patterns of the expression. The difficulty in detecting such modules and recovering the latent factors lies in the high dimensionality of the data, and the lack of knowledge in module membership.

**Methods:**

Here we describe a method based on community detection in the co-expression network. It consists of inference-based network construction, module detection, and interacting latent factor detection from modules.

**Results:**

In simulations, the method outperformed projection-based modular latent factor discovery when the input signals were not Gaussian. We also demonstrate the method's value in real data analysis.

**Conclusions:**

The new method nMLSA (network-based modular latent structure analysis) is effective in detecting latent structures, and is easy to extend to non-linear cases. The method is available as R code at http://web1.sph.emory.edu/users/tyu8/nMLSA/.

## Background

Modularity is a common characteristic of high-throughput biological data [1]. In a large system, the biological units, i.e. features (genes, proteins, or metabolites) are organized into quasi-autonomous modules. In expression data, each expression module can be modeled reasonably well using the latent factor approach [2,3]. Given the involvement of thousands of features, an unknown number of modules, and unknown module membership of the features, it is difficult to faithfully detect the modules and recover the underlying latent factors controlling the modules.

Dimension reduction methods at the global level, such as Principal Component Analysis (PCA), Independent Component Analysis (ICA) [4], sparse PCA [5,6], and Bayesian decomposition [7] are not effective in detecting localized signals. Clustering methods group co-expressed features together [8], which may help identify modules that are controlled by a single underlying signal [9,10]. However in real data, the features involved in the same module may not co-express when more than one latent factors control the module. We previously proposed the projection-based Modular Latent Structure Analysis (MLSA) [11], which detects modules using iteratively re-weighted singular value decomposition (SVD). So far there are no other modular decomposition methods. In this study, we seek to improve the method using a totally different approach. Our goal is to develop a method that is more intuitive, flexible, and involves less *ad hoc *parameter choices.

Using networks constructed from expression data can provide a flexible framework for module detection [12-14]. Here we present a method to identify modules and the underlying latent signals in three steps: (1) constructing a co-expression network based on statistical inference and local false discovery rate (lfdr); (2) detecting communities in the network; and (3) recovering interacting latent factors from the modules.

The goal of the algorithm is to achieve modular matrix decomposition. We attempt to solve the problem by assembling tools from some well-established fields. The first is the reverse engineering of genome-scale networks. There are a number of methods available in this area, which were designed with different objectives, including Gaussian Graphical Models where the absence of an edge signifies conditional independence [15,16], co-expression network where edges signify marginal dependence [13], information theory-based networks [17], and Bayesian networks [18]. In this study, we designed our own method to estimate an inference-based co-expression network using the local false discovery rate (lfdr) concept [19-21]. The use of local fdr makes the procedure adaptive to shifts of baseline correlation levels and avoids constructing overly dense networks when there are pervasive low-level correlations between genes. Once the network is constructed, we borrow a method from the mature field of community detection in large networks [22-25]. This is followed by latent factor extraction and rotation using factor analysis methods [26]. Added together, the assembled tools make a very good heuristic solution to the modular decomposition problem.

We demonstrate the superiority of the new method against existing modular and global decomposition methods using simulations, and apply the method to a real dataset to show it detects biologically meaningful modules that are controlled by multiple latent factors.

## Methods

### The objective

Given a data matrix ***G****_p×n _*with *p *features measured in *n *conditions, we seek to assign subgroups of the features into modules, such that within each module, the expression levels of the features can be modeled by a linear factor model

$$G_{q\times n}^{\left(module\right)}=L_{q\times r}F_{r\times n}+E_{q\times n},$$

where *q *is the size of the module, *r *is the number of latent factors controlling the module, ***L ***is the regulation strength (loading) matrix, and ***E ***is the residual matrix. Our interest is estimating (1) the number of modules, (2) the module membership of the features, (3) the activities of the latent factors controlling each module (***F ***matrix), and (4) the regulation strength of each factor on each feature (***L ***matrix).

### The estimation procedure

**Figure 1:**
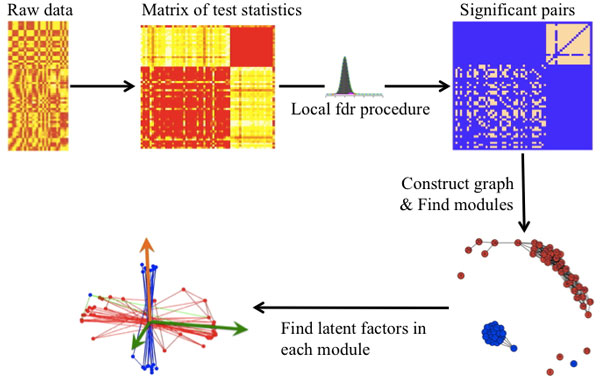
**An illustration of the procedure using a toy example dataset**. The features belong to two modules. One module is controlled by a single factor, and the other controlled by two factors.

Figure 1 illustrates the procedure using a toy dataset with two modules. Generally, three steps are involved.

*Step 1. Constructing co-expression network based on local fdr*. We use the concept of local false discovery rate (lfdr) to establish links between features [19]. First, we compute the correlation coefficients $r_{ij}$ between all pairs of features. Secondly, we transform the correlation coefficients by

$$t_{ij}=r_{ij}\sqrt{\left(n-2\right)/\left(1-r_{ij}^{2}\right)},$$

so that the distribution of the resulting statistic is close to normal under the null hypothesis that the pair of features are independent [27]. Thirdly, we compute the local false discovery rate using Efron's procedure [19]. The local fdr is a statistical statement of how likely two features are independent given we observe the statistics from all pairs of features. Fourth, if the local fdr value for a pair of features is smaller than a threshold, *e.g*. 0.2, an edge is established between the two features.

*Step 2. Module detection in the co-expression network*. We first use a well-established method that detects dense sub-graphs from a sparse graph by short random walks [25]. To fine-tune the results, we conduct an additional community-merging step. For a pair of communities $C_{i}$ that contains $m_{i}$ features and $k_{i}$ within-community connections, and $C_{j}$ that contains $m_{j}$ features and $k_{j}$ within-community connections, we divide the number of between-community connections $k_{ij}$ by the expected number of connections if the communities were indeed one

$$\delta_{ij}=\frac{k_{ij}}{m_{i}m_{j}\times \left(k_{i}+k_{j}\right)}\left(\left(\begin{array}{c} m_{i}\\ 2 \end{array}\right)+\left(\begin{array}{c} m_{j}\\ 2 \end{array}\right)\right).$$

We then pool all the $\delta_{ij}$ values computed from all pairs of communities and examine the distribution. Any outlier $\delta_{ij}$, defined by a value higher than the median plus four times the difference between the 75^th ^percentile and the median, signifies a community pair that should be merged into a single community.

*Step 3. Detecting latent factors from each module*. For each module, we first conduct an eigenvalue decomposition of the covariance matrix, and select all eigenvectors that account for at least 5% of the data variance. We then find the projection length of each feature onto each eigenvector ${\left\{l_{i}^{\left(j\right)}\right\}}_{i=1,\ldots m_{i},j=1,\ldots ,n_{j}}$, where *i *denotes the feature and *j *denotes the eigenvector. The value $m_{j}$ is the number of features in the module, and $n_{j}$ is the number of eigenvectors under consideration.

Two eigenvectors are considered "interactive" if the correlation of the projection length of the features onto these two vectors is statistically significant. We initiate a selected vector set with only the first eigenvector. Then from the second eigenvector on, if the eigenvector is interactive with any vector in the selected set, it is added to the selected set. Otherwise we stop the iteration and return the selected vector set as the latent variables of the module. If more than one eigenvector is selected, we rotate them using oblique rotation [26].

*Step 4. The overall factor model*. After finding a collection of ***F ***matrices, we can combine them into an overall factor model with a sparse loading matrix to interpret the gene expression. Let *K *be the total number of latent factors found, ***B ***be the combined factor activity matrix of all the factor scores, ***L ***be the loading matrix, and ***E ***be the unexplained expression, we have a factor model,

$$G_{p\times n}=L_{p\times K}B_{K\times n}+E_{p\times n}$$

The values in ***L ***can be filled in two ways. The first is by performing linear regression of each gene against only the factors of the modules the gene is assigned to. Alternatively, we can perform regularized regression of each gene against all the factors using lasso [28] with BIC (Bayesian information criterion) model selection.

### Simulation study

We refer to our method as "Network-based Modular Latent Factor Analysis (nMLSA)". We compared the method with MLSA [11], PCA, ICA [29], factor analysis with oblique rotation [26], gene shaving [9], and sparse principal component analysis (SPCA) [5]. In each simulation, we generated a gene expression dataset with 10 modules. Every module consisted of 100 simulated genes. The number of latent factors controlling the module was randomly selected between 1 and 3. An additional 1000 pure noise genes were generated from the standard Gaussian distribution. We vary the following parameters in the simulations:

(1) The latent factor scores were either independent Gaussian, or randomly chosen from a mixture of four types: Gaussian, sine wave, square wave, and sawtooth wave (Additional file 1 Figure S1). The setting stayed the same for every module in each simulated dataset.

(2) Different levels of within-module loading sparsity, i.e. proportion of zero loadings, were tested. The sparsity of the loading matrix was achieved by drawing samples from the binomial distribution. After the non-zero positions in the loading matrix was determined, for every simulated gene, if there were *m *controlling factors, we divided [0, 1]into *m *regions by drawing *(m-1) *samples from the uniform distribution between 0 and 1. We then used the sizes of the regions as the loadings for the gene. Half of the loadings were then multiplied by -1 to generate negative loadings. The sparsity levels tested were 0%, 30% and 60%. The setting stayed the same for every module in each simulated dataset.

(3) After multiplying the loading matrix and the factor score matrix to generate the simulated expression matrix, Gaussian random noise was added to achieve different signal to noise ratios (values used: 1, 2). The setting stayed the same for every module in each simulated dataset.

The number of samples was set at 100. All possible combinations of the parameters were tested, each repeated 100 times.

To judge the performance of the methods, we used the information of the true hidden factors to group the identified factors. Let *K *be the combined hidden factor count from all modules in the simulated dataset. We first performed linear regression of every identified factor against each hidden factor group (those controlled the same module), and recorded the multiple R^2^. The identified factor was assigned to the hidden factor group with which it had the largest R^2 ^value. The *K *identified factors with the largest R^2 ^values were retained for the next step. Second, we performed linear regression of every true hidden factor against the identified factors assigned to its group, and recorded the multiple R^2 ^as the level of recovery of the true hidden factor. The ideal method should yield multiple R^2 ^values close to one. After repeating the simulation from every parameter setting 100 times, we compared the methods by the distribution of the multiple R^2 ^values.

## Results

### Simulation results

**Figure 2:**
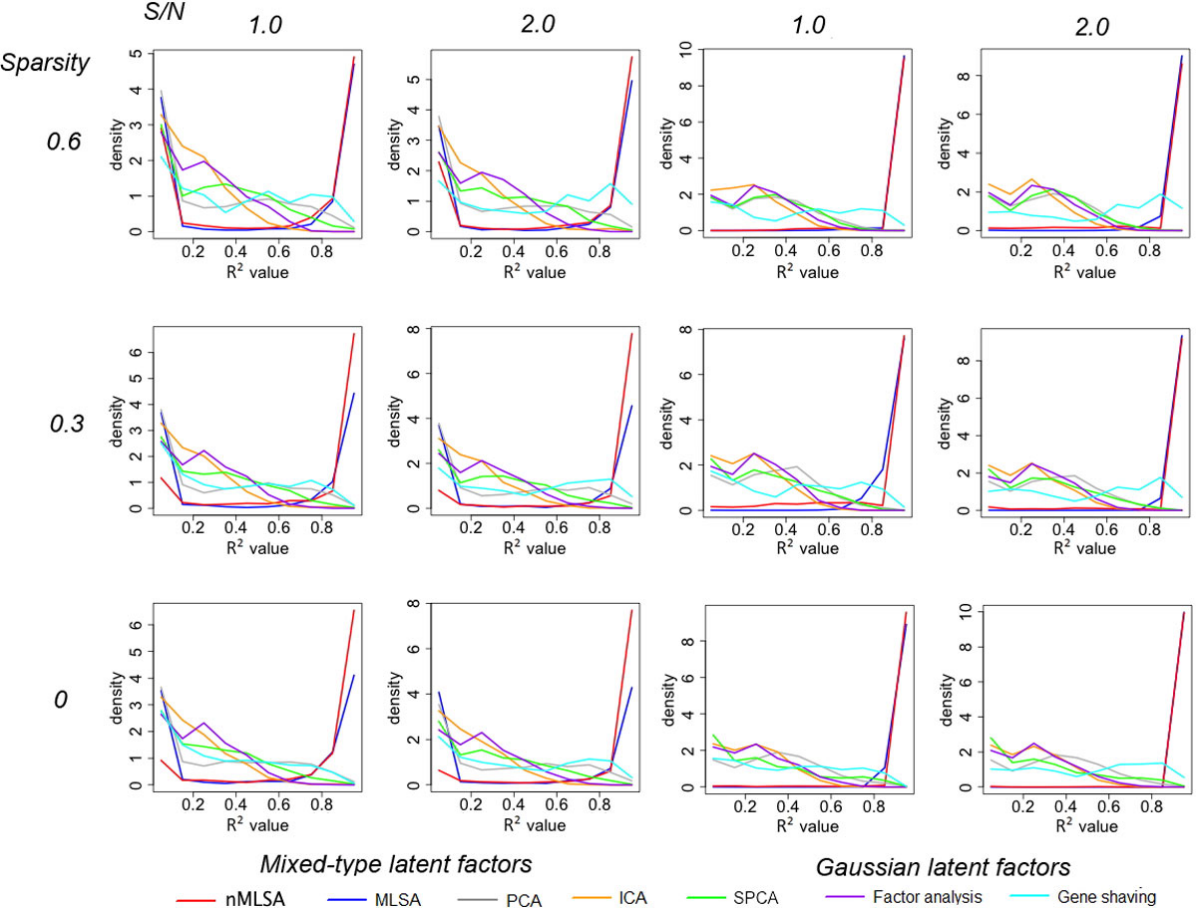
**Simulation results from modular latent structure models**. In every simulation, 10 modules, each consisting of 100 simulated genes, were generated. The number of latent factors per module was randomly selected between 1 and 3. The latent factors were either independent Gaussian (two right columns), or randomly chosen from a mixture of four types (two left columns). Gaussian random noise was added to achieve different signal to noise ratios (columns), and different levels of within-module sparsity (proportion of zero loadings) were tested (rows). An additional 1000 pure noise genes were generated from the standard Gaussian distribution. Each simulation setting was repeated 100 times. The success of latent factor recovery was evaluated by the R^2 ^values obtained by the regression of each latent factor against the identified factors assigned to the module to which the latent factor belongs. The relative frequencies (10 equal-sized bins between 0 and 1, equivalent to the histogram) of the R^2 ^values are plotted.

The simulation results are summarized in Figure 2. Each sub-plot represents a parameter setting. The relative frequencies (10 equal-sized bins between 0 and 1, equivalent to the histogram) of the R^2 ^values are plotted in Figure 1. Different colors represent different methods. The curves are effectively histograms of the multiple R^2 ^values. The curve of a better method should show higher frequency in larger R^2 ^values. In all the scenarios, clearly nMLSA (red) and MLSA (blue) outperformed the other methods.

When the true signals were Gaussian (Figure 2; two right columns), nMLSA and MLSA yielded similar results. Both methods recovered the hidden factors almost perfectly in all sparsity (rows) and noise (columns) settings. When the true signals were randomly drawn from four different types (Figure 2; two left columns), nMLSA outperformed MLSA. Both methods tend to either fully recover or totally miss a hidden factor, as indicated by spikes at R^2 ^= 1 and R^2 ^= 0. However when the within-module sparsity was moderate to low (30% and 0%), nMLSA showed a roughly 3-fold reduction in the chance to miss hidden factors, and accordingly a much higher chance to faithfully recover the hidden factors.

### Real data analysis

**Figure 3:**
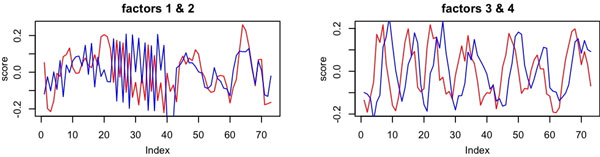
**The top four factors from the Spellman dataset form two modules**. On the x-axis the four time series are displayed in sequential order.

The Spellman cell cycle data consists of four time-series, each covering roughly two cell cycles [30]. The array data consists of 73 conditions and 6178 genes. Because of phase differences, the cell cycle-related genes cannot be easily summarized by clusters although many of them exhibit periodic patterns [31]. We applied nMLSA to the cell cycle data as a whole, in order to discover common patterns across the four time series. Our method identified 7 modules containing 10 latent factors in total. The two largest modules each contained two latent factors (Figure 3).

While MLSA also detected the second module, it failed to detect module 1 found by nMLSA (Figure 3, left panel). Functional analyses using Gene Ontology [32] indicate the module is highly biologically meaningful. Based on hypergeometric tests using the GOStats package [33], genes associated with the first factor of the module strongly over-represent biological processes related to RNA processing and the ribosome, which is central to protein biosynthesis (Table 1). Genes associated with the second factor over-represent biological processes related to protein degradation, transport and localization (Table 2). Protein transport and localization processes are naturally coordinated with protein biosynthesis. Evidences also point to the co-regulation of protein biosynthesis and protein degradation, under normal circumstances and experimental interference [34-36].

**Table 1:** Top 25 GO terms overrepresented by genes associated with factor 1.

GOBPID	Pvalue	Term
GO:0042254	3.27E-75	ribosome biogenesis
GO:0022613	2.04E-68	ribonucleoprotein complex biogenesis
GO:0034470	6.20E-67	ncRNA processing
GO:0034660	1.31E-65	ncRNA metabolic process
GO:0006364	1.55E-60	rRNA processing
GO:0016072	1.47E-59	rRNA metabolic process
GO:0006396	3.00E-51	RNA processing
GO:0071843	1.48E-50	cellular component biogenesis at cellular level
GO:0042273	5.74E-33	ribosomal large subunit biogenesis
GO:0000460	4.04E-30	maturation of 5.8S rRNA
GO:0000466	2.05E-29	maturation of 5.8S rRNA from tricistronic rRNA transcript (SSU-rRNA, 5.8S rRNA, LSU-rRNA)
GO:0016070	1.33E-26	RNA metabolic process
GO:0042274	4.46E-26	ribosomal small subunit biogenesis
GO:0044085	3.25E-25	cellular component biogenesis
GO:0030490	1.57E-21	maturation of SSU-rRNA
GO:0000462	3.11E-21	maturation of SSU-rRNA from tricistronic rRNA transcript (SSU-rRNA, 5.8S rRNA, LSU-rRNA)
GO:0000469	3.59E-21	cleavage involved in rRNA processing
GO:0010467	7.58E-21	gene expression
GO:0090304	2.29E-16	nucleic acid metabolic process
GO:0009451	2.58E-16	RNA modification
GO:0000478	8.00E-16	endonucleolytic cleavage involved in rRNA processing
GO:0000479	8.00E-16	endonucleolytic cleavage of tricistronic rRNA transcript (SSU-rRNA, 5.8S rRNA, LSU-rRNA)
GO:0006139	1.09E-15	nucleobase-containing compound metabolic process
GO:0000447	1.37E-15	endonucleolytic cleavage in ITS1 to separate SSU-rRNA from 5.8S rRNA and LSU-rRNA from tricistronic rRNA transcript (SSU-rRNA, 5.8S rRNA, LSU-rRNA)
GO:0000472	1.42E-14	endonucleolytic cleavage to generate mature 5'-end of SSU-rRNA from (SSU-rRNA, 5.8S rRNA, LSU-rRNA)

**Table 2:** Top 25 GO terms overrepresented by genes associated with factor 2.

GOBPID	Pvalue	Term
GO:0006511	1.99E-09	ubiquitin-dependent protein catabolic process
GO:0019941	2.44E-09	modification-dependent protein catabolic process
GO:0044257	2.65E-09	cellular protein catabolic process
GO:0051603	3.79E-09	proteolysis involved in cellular protein catabolic process
GO:0043632	4.21E-09	modification-dependent macromolecule catabolic process
GO:0030163	7.26E-09	protein catabolic process
GO:0010499	2.24E-08	proteasomal ubiquitin-independent protein catabolic process
GO:0044265	9.94E-08	cellular macromolecule catabolic process
GO:0007005	1.38E-07	mitochondrion organization
GO:0043623	2.59E-07	cellular protein complex assembly
GO:0043248	3.56E-07	proteasome assembly
GO:0009057	5.19E-07	macromolecule catabolic process
GO:0006508	5.25E-07	proteolysis
GO:0071842	3.02E-06	cellular component organization at cellular level
GO:0015031	4.89E-06	protein transport
GO:0008104	6.28E-06	protein localization
GO:0043161	6.81E-06	proteasomal ubiquitin-dependent protein catabolic process
GO:0045184	7.86E-06	establishment of protein localization
GO:0010498	1.01E-05	proteasomal protein catabolic process
GO:0006461	1.35E-05	protein complex assembly
GO:0034613	1.41E-05	cellular protein localization
GO:0070271	2.51E-05	protein complex biogenesis
GO:0070727	2.56E-05	cellular macromolecule localization
GO:0034621	3.00E-05	cellular macromolecular complex subunit organization
GO:0009987	3.93E-05	cellular process

**Figure 4:**
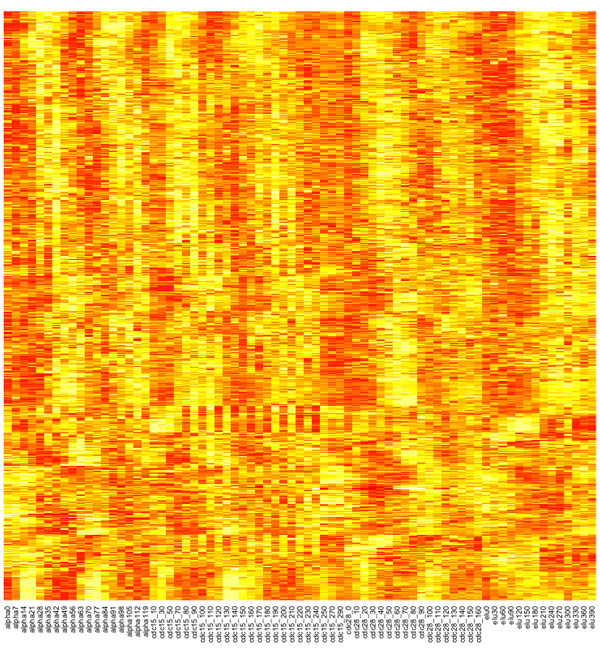
**Expression levels of genes involved in module 2**. Genes are hierarchically clustered. The four time series are displayed in sequential order.

The second module is even more intuitive biologically. The factor scores showed that the second module was governed by two periodic latent factors with similar periodicity but different phases (Figure 3, right). Genes of this module showed clear periodic behavior with different phase shifts (Figure 4), which is consistent with the biological knowledge that cell-cycle genes are activated at different phases of the cell cycle [30]. We analyzed the functionalities of the genes associated with each factor using gene ontology (GO). It was clear that cell cycle-related biological processes dominated the list of top processes overrepresented by genes associated with either latent factors (Tables 3 &4). Other methods used in the simulations, except MLSA, could not group cell cycle genes with different phase shift into a single module.

**Table 3:** Top 25 GO terms overrepresented by genes associated with factor 3.

GOBPID	Pvalue	Term
GO:0000278	4.02E-19	mitotic cell cycle
GO:0022402	4.08E-17	cell cycle process
GO:0007049	6.05E-17	cell cycle
GO:0022403	5.72E-15	cell cycle phase
GO:0007017	8.75E-15	microtubule-based process
GO:0048285	2.31E-13	organelle fission
GO:0000280	5.08E-13	nuclear division
GO:0051301	8.45E-13	cell division
GO:0000226	8.50E-13	microtubule cytoskeleton organization
GO:0000087	9.22E-13	M phase of mitotic cell cycle
GO:0007067	1.39E-12	mitosis
GO:0000279	2.71E-09	M phase
GO:0007010	1.75E-08	cytoskeleton organization
GO:0007059	2.33E-08	chromosome segregation
GO:0030472	3.11E-08	mitotic spindle organization in nucleus
GO:0032886	3.46E-08	regulation of microtubule-based process
GO:0070507	3.46E-08	regulation of microtubule cytoskeleton organization
GO:0016043	5.99E-08	cellular component organization
GO:0007051	7.48E-08	spindle organization
GO:0051329	7.69E-08	interphase of mitotic cell cycle
GO:0007052	1.09E-07	mitotic spindle organization
GO:0051325	1.12E-07	interphase
GO:0006928	1.15E-07	cellular component movement
GO:0007018	2.33E-07	microtubule-based movement
GO:0010564	6.27E-07	regulation of cell cycle process

**Table 4:** Top 25 GO terms overrepresented by genes associated with factor 4.

GOBPID	Pvalue	Term
GO:0007049	5.54E-08	cell cycle
GO:0051301	8.21E-08	cell division
GO:0000278	7.99E-07	mitotic cell cycle
GO:0000087	1.12E-06	M phase of mitotic cell cycle
GO:0022402	1.57E-06	cell cycle process
GO:0022403	6.92E-06	cell cycle phase
GO:0048285	2.16E-05	organelle fission
GO:0000280	3.03E-05	nuclear division
GO:0010458	3.55E-05	exit from mitosis
GO:0000910	3.70E-05	Cytokinesis
GO:0000279	4.43E-05	M phase
GO:0007067	7.91E-05	Mitosis
GO:0033205	0.000523865	cell cycle cytokinesis
GO:0032506	0.000572574	cytokinetic process
GO:0010970	0.001012686	microtubule-based transport
GO:0030473	0.001012686	nuclear migration along microtubule
GO:0030705	0.001012686	cytoskeleton-dependent intracellular transport
GO:0072384	0.001012686	organelle transport along microtubule
GO:0000114	0.00177365	regulation of transcription involved in G1 phase of mitotic cell cycle
GO:0046459	0.00185316	short-chain fatty acid metabolic process
GO:0007018	0.002062712	microtubule-based movement
GO:0016575	0.002062712	histone deacetylation
GO:0032392	0.002399826	DNA geometric change
GO:0010696	0.002438214	positive regulation of spindle pole body separation
GO:0007097	0.002544351	nuclear migration

## Discussions

In this study, we developed the network-based modular latent structure analysis (nMLSA). It is aimed at detecting expression modules and latent factors controlling the modules, the same goal as the original MLSA [11]. Compared to MLSA, the new method is based on a totally different setup, and is substantially advantageous. Firstly, the number of tuning parameters and heuristic choices is substantially less compared to MLSA. Secondly, the method is much more intuitive to understand. Thirdly, it is more flexible. As an example, one can easily limit the gene relations to positive correlations and ignore negative correlations using nMLSA, while MLSA has to take both positive and negative correlations. Fourth, nMLSA can be adapted for nonlinearly associated modules if a nonlinear association measure is used in the co-expression network building, while MLSA is limited to linear relations. In the nonlinear case, it is difficult to define latent factors. The challenge is subject to our future studies.

Instead of using hard cutoffs, nMLSA utilizes the concept of local false discovery rate (lfdr). As different datasets exhibit different levels of baseline correlation [37], using hard cutoffs on correlations may result in unsatisfactory results. Using local false discovery rate procedures that are flexible in the null distribution estimation, nMLSA is naturally adaptive to the characteristics of the data. Given the nMLSA procedure relies on existing network community detection algorithms, it is admitted that the performance of the method relies on the choice of the community detection algorithm. The research field of community detection is mature and a number of good methods are available. Thus it is not difficult to tune the method to achieve good performance.

## Conclusions

In summary, the new network-based method nMLSA is more effective than existing methods in recovering biologically meaningful latent variables and latent variable groups. The method can potentially be extended to detect nonlinearly associated modules if a nonlinear association measure is used to build the network.

## Competing interests

None.

## Authors' contributions

TY developed the computational method, conducted simulations. TY and YB conducted real data analyses, interpreted the results, and drafted the manuscript.

## Supplementary Material

Additional file 1 **Figure S1**. The four types of input signal from which the data were simulated. Click here for file
