# Supplementary figures and images for: Network-based modular latent structure analysis
Source: BMC Bioinformatics. 2014 Nov 13;15(Suppl 13):S6. doi: 10.1186/1471-2105-15-S13-S6 (PMC4248660; doi:10.1186/1471-2105-15-S13-S6)

Supporting Material

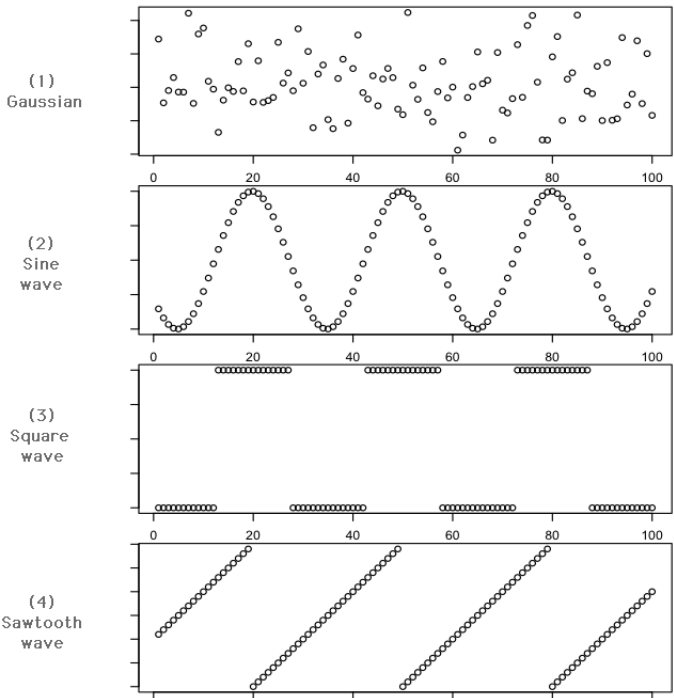

**Figure S1.** The four types of input signal from which the data were simulated.

Supplement: Additional file 1 — Figure S1. The four types of input signal from which the data were simulated. [file 1471-2105-15-S13-S6-S1.pdf]
